# Some Remarks on the Medicinal Properties of Ledum Palustre

**Published:** 1800-08

**Authors:** William George Maton


### *Some Remarks on the Medicinal Properties of Ledum Palustre.*

By WILLIAM GEORGE MATON, M.B. F.R.S. &c.

NOTWITHSTANDING the multitude and variety of the articles already received into the Materia Medica, and the confequent hefitation with which cautious practitioners adopt the exhibition of lefs noted medicines, the healing art cannot furely be provided with too many refources; nor will the fcientific phyfician ceafe to feek in the treafures of Nature for new fubftances fubfervient to his profeffion. But, without prefuming to recommend any fubftances which have very recently been difcovered to poffefs medical properties, I wifh only to bring into general notice, fome which have long been known, but but which do not ſeem to have been ſubjected to experiment in this country.

Of the diſeaſes which moſt obſtinately reſiſt the ordinary means of cure, none are leſs productive of credit to the practitioner, or more haraſſing to the patient, than thoſe which are commonly called *cutaneous*; and in ſuch caſes it may be worth while to try the effects of medicines ever ſo partially recommended. A very ingenious foreign phyſician has lately informed me, that a chronic diſeaſe of the impetiginous claſs, which had reſiſted various other remedies, was at length completely cured by an infuſion of Ledum paluſtre taken internally. This fact did not appear ſurpriſing, when I recollected that the ſame plant had been recommended by the Swedes for the cure of Pſora. In the 8th vol. of the *Amœnitates Academicæ*, edited by Schreber, Dr. Weſtring adviſes the uſe of the infuſion as a bath. Profeſſor Pallas ſtates, that the ſame remedy is employed by the Ruſſians. A Swediſh phyſician, of the name of Wahlbom, cured a moſt obſtinate caſe of tinea by repeated waſhings with a decoction of the Ledum. Of the general aſtringent properties of the plant, I find accounts ſcattered in ſeveral writers. In Hungary it is uſed as a gargariſm in ulcerations of the throat; and it has been much extolled by Scopoli for its utility in ſuch caſes. Weſtring's Diſſertation, before mentioned, informs us, that it manifeſted a moſt decided efficacy in an epidemical angina, which was of a malignant nature; but by means of gargariſms and cataplaſms of the Ledum, yielded very ſpeedily. Jacquin records ſimilar ſucceſs attending its exhibition for diſeaſes of the throat among the inhabitants of the Carpathian mountains. Theſe evidences, however, relate principally to the external uſe of Ledum paluſtre. The exhibition of it internally is not without conſiderable teſtimony in its favour. Falk mentions, that in ſome parts of the Ruſſian empire, it is not an unfrequent remedy for intermittents. But what would lead me to form a ſtill higher idea of the value of this vegetable, is a fact recorded by the celebrated Linnæus, in his Travels to Weſtrogothia. He ſays, that the common people in that province cure Pertuſis by means of the infuſion. It was through the great naturaliſt, probably, that the medicine obtained a place in the Pharmacopeia of the Swedes, among whom, I underſtand, it is very generally uſed. Roſenſtein (in his Diſeaſes of Children) recommends it for the hooping cough; but it is alſo employed as a remedy for diarrhœa and dyſentery among his countrymen.

Theſe obſervations, to which I only regret that I have nothing to add founded on any experience of my own, may be ſufficient perhaps to induce Engliſh practitioners to make trial of of a plant which would feem to poffefs very active properties. Its very odour indicates an aromatic quality, though this is not fo ftrong in the fpring as during the ftate of florefcence. Wef-tring mentions a curious fact refpecting the permanence of the aroma. A dried fpecimen, which he found in the Burfe-rian Herbarium at Upfal, retained a very fenfible fragrance, though upwards of 150 years had elapfed from the time of its being gathered. An infufion of the leaves is fomewhat bitter, and conveys a fenfation to the palate fimilar to that produced by turpentine; a good evidence of its poffeffing an aftringency. There is undoubtedly a narcotic quality in a ftrong decoction of the plant, fome of the northern nations being accuftomed to boil fticks of it in beer, for the fake of imparting to the latter an inebriating effect; and this liquor has been obferved to occafion violent vomitting, cephalalgia, and even fyncope. Some caution, therefore, is neceffary in the exhibition of the vegetable; and though in many cafes it may be advifeable to keep up that ftate, refembling ftupor, by which the advantage-ous effects of cicuta and other narcotics are produced, the dofe ought to be fmall at firft. Of the recent plant, four ounces may be infufed in a quart of boiling water, and two ounces of the ftrained liquor are fufficient for the firft exhibition.

Ledum paluftre grows fo abundantly in all the northern parts of the continent, that large quantities may be obtained without difficulty; though, as its favourite foil is of a moift, boggy nature, and as it is found on the declivities of lofty mountains, the cultivation of this plant may not often prove very fuccefsful. No animals appear to be fond of it, excepting the goat, which Linnæus tells us, (in the Pan Succus) will often brouze it; and there is a fpecies of phalæna that dwells on its leaves; but infects in general fhun, and are fo much af-fected by its odour, that the fmoke is ufed for the extermina-tion of lice and other vermin. The fpirit obtained by diftilla-tion would feem likely to be moft efficacious for this purpofe.

There is another fpecies of Ledum (fcarcely deferving, how-ever, to be confidered as different) which grows very plenti-fully in the vicinity of Hudfon's Bay, in North America, and is generally known by the name of *Labrador Tea*, an appella-tion that indicates its being ufed as a beverage by the natives of that diftrict, probably on account of this plant poffeffing qualities fimilar to thofe of Ledum paluftre,—I mean more particularly, that of intoxicating. Ledum latifolium, as the name implies, is diftinguifhed by its broader leaves; but in no other refpect does it appear to differ from its congener. I have tafted infufions of the dried leaves, and found the flavour the fame. This plant is well defcribed by Jacquin in his *Collecta-*

*nea*,

*nea*, tom. ii. and in the new edition of the *Species Plantarum*, by Wildenow, diftinguifhed thus, *Ledum (latifolium) foliis oblongis, margine revolutis, subtus tomentoris floribus subpentandris*, tom. ii. p. 602.

P. S. Since the above was written, I have found in the Tranfactions of the Academy of Sciences of Stockholm, (1774) an account of the efficacy of Ledum paluftre as a remedy for Lepra. Dr. Odhelius fpeaks of his fuccefs in the exhibition of it with the higheft fatisfaction.

W. G. M.

*Craven Street, July* 3, 1800.

